# Methyl 9-(4-meth­oxy­phen­yl)-19-methyl-3,12-di­aza­penta­cyclo­[10.7.0.0^2,10^.0^3,8^.0^13,18^]nona­deca-1(19),13(18),14,16-tetra­ene-10-carboxyl­ate

**DOI:** 10.1107/S1600536813010489

**Published:** 2013-04-20

**Authors:** S. Selvanayagam, B. Sridhar, S. Kathiravan, R. Raghunathan

**Affiliations:** a Department of Physics, Kalasalingam University, Krishnankoil 626 126, India; b Laboratory of X-ray Crystallography, Indian Institute of Chemical Technology, Hyderabad 500 007, India; c Department of Organic Chemistry, University of Madras, Guindy Campus, Chennai 600 025, India

## Abstract

The title ester, C_27_H_30_N_2_O_3_, crystallizes with two independent mol­ecules in the asymmetric unit whose geometrical features are similar. In each mol­ecule, the pyrrolidine ring adopts an envelope conformation, with the fused C atom shared with the piperidine ring as the flap, and the piperidine ring adopts a chair conformation. In the crystal, C—H⋯π inter­actions link the inversion-related molecules and form a dimeric arrangement in the unit cell.

## Related literature

For the superposition of mol­ecules using *Qmol*, see: Gans & Shalloway (2001 ▶). For ring-puckering parameters, see: Cremer & Pople (1975 ▶).
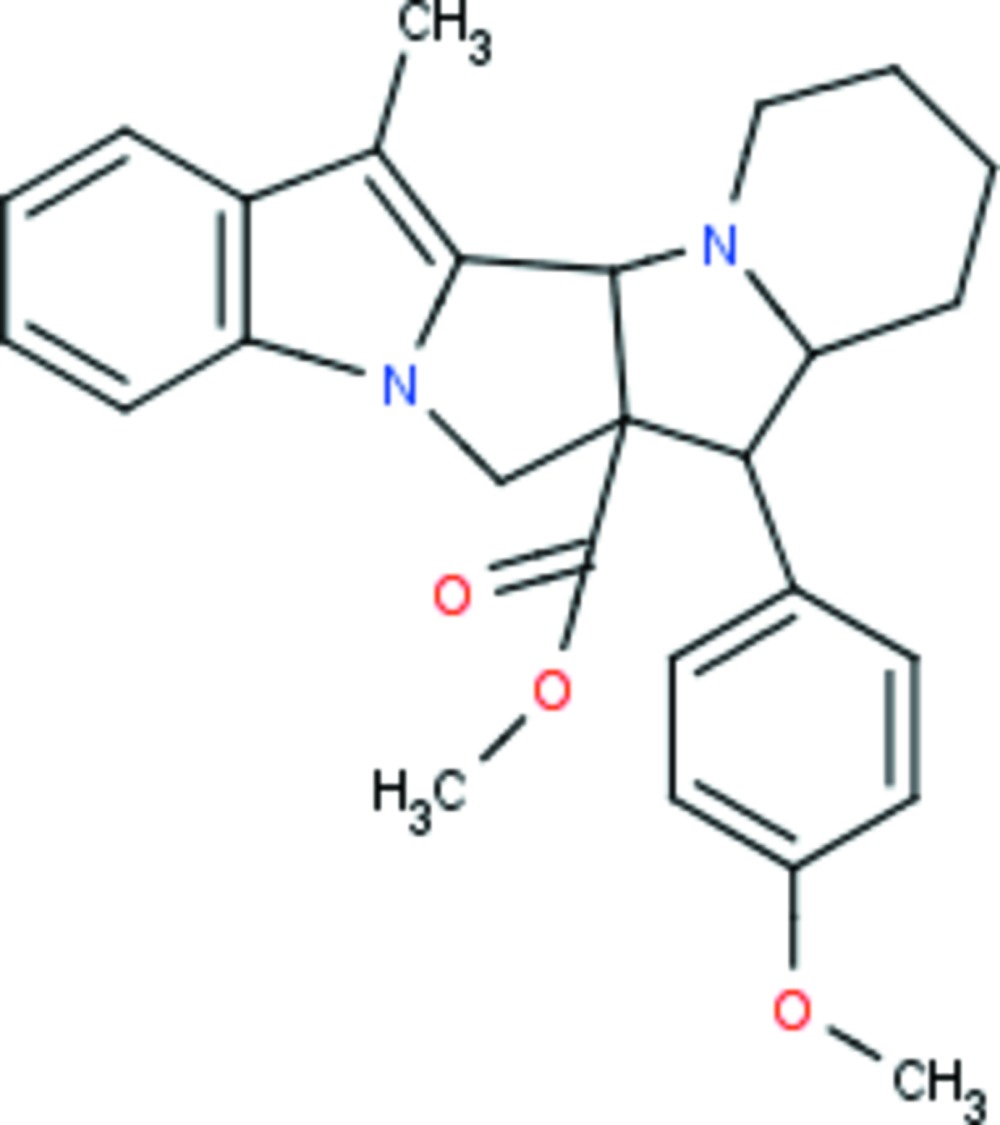


## Experimental

### 

#### Crystal data

C_27_H_30_N_2_O_3_
*M* *_r_* = 430.53
Monoclinic, 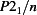

*a* = 22.672 (3) Å
*b* = 8.8049 (11) Å
*c* = 24.431 (3) Å
β = 110.487 (2)°
*V* = 4568.6 (10) Å^3^
*Z* = 8
Mo *K*α radiation
μ = 0.08 mm^−1^
*T* = 292 K
0.22 × 0.20 × 0.18 mm

#### Data collection

Bruker SMART APEX CCD area-detector diffractometer
42435 measured reflections
8045 independent reflections
5762 reflections with *I* > 2σ(*I*)
*R*_int_ = 0.069

#### Refinement

*R*[*F* ^2^ > 2σ(*F* ^2^)] = 0.092
*wR*(*F* ^2^) = 0.182
*S* = 1.18
8045 reflections
583 parameters
H-atom parameters constrained
Δρ_max_ = 0.43 e Å^−3^
Δρ_min_ = −0.16 e Å^−3^

### 

Data collection: *SMART* (Bruker, 2001 ▶); cell refinement: *SAINT* (Bruker, 2001 ▶); data reduction: *SAINT*; program(s) used to solve structure: *SHELXS97* (Sheldrick, 2008 ▶); program(s) used to refine structure: *SHELXL97* (Sheldrick, 2008 ▶); molecular graphics: *ORTEP-3 for Windows* (Farrugia, 2012 ▶) and *PLATON* (Spek, 2009 ▶); software used to prepare material for publication: *SHELXL97* and *PLATON* (Spek, 2009 ▶).

## Supplementary Material

Crystal structure: contains datablock(s) I, global. DOI: 10.1107/S1600536813010489/ng5324sup1.cif

Structure factors: contains datablock(s) I. DOI: 10.1107/S1600536813010489/ng5324Isup2.hkl

Supplementary material file. DOI: 10.1107/S1600536813010489/ng5324Isup3.cml

Additional supplementary materials:  crystallographic information; 3D view; checkCIF report

## Figures and Tables

**Table 1:** Hydrogen-bond geometry (Å, °) *Cg*1 and *Cg*2 are the centroids of the C19*A*–C24*A* and C19*B*–C24*B* rings, respectively.

*D*—H⋯*A*	*D*—H	H⋯*A*	*D*⋯*A*	*D*—H⋯*A*
C25*A*—H25*A*⋯*Cg*1^i^	0.96	2.81	3.606 (5)	141
C25*B*—H25*F*⋯*Cg*2^ii^	0.96	2.68	3.495 (5)	143

Symmetry codes: (i) 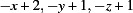; (ii) 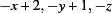.

**Fig. 1.**
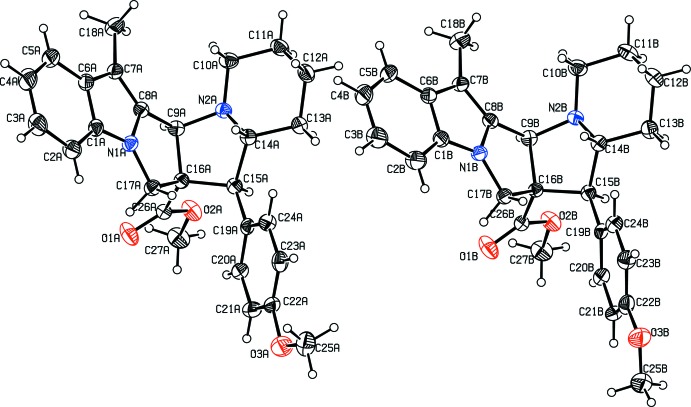
The molecular structure of the title compound, showing the atom-numbering scheme. Displacement ellipsoids are drawn at the 30% probability level

**Fig. 2.**
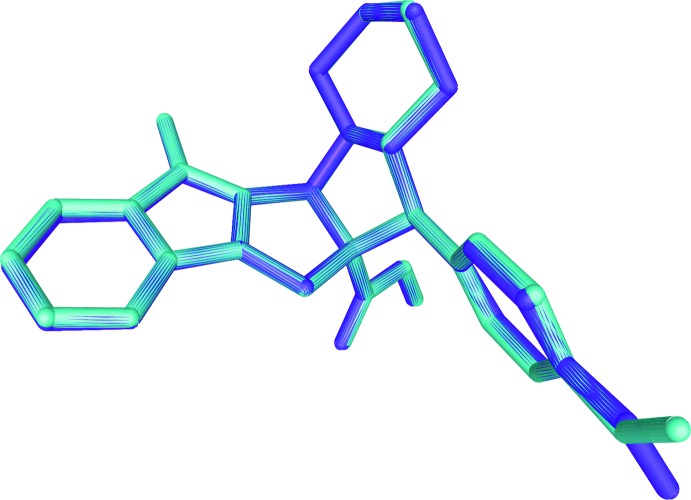
Superposition of molecule A (cyan) with the molecule B (pink) in (I).

## supplementary crystallographic information

### Comment

In continuation of our work on the crystal structure analyis of pyrrolidine derivatives, we have undertaken the crystal structure determination of the title compound, and the results are presented here.

The X-ray study confirmed the molecular structure and atomic connectivity for (I), as illustrated in Fig. 1. The asymmetric unit of (I) contains two molecules (Fig. 1); their corresponding bond lengths and bond angles are in good agreement. Fig. 2 shows a superposition of the central pyrrolidine ring of both the molecules using Qmol (Gans & Shalloway, 2001); the r.m.s. deviation is 0.012 Å.

The phenyl ring and the first two five membered rings are planar with the maximum deviation of -0.135 (3) and -0.178 (3) Å for atom C16 in molecules A and B, respectively.

The pyrrolidine ring (third five membered ring) is in an envelope conformation, with puckering parameters (Cremer & Pople, 1975) q_2_ = 0.464 (3) Å and φ = 73.0 (4) ° for molecule A, and q_2_ = 0.455 (4) Å and φ = 75.7 (4) ° for molecule B. Atom C14 deviates 0.697 (3) and 0.697 (2) Å from the least-squares plane through the remaining four atoms (C9/N2/C15/C16) of that of ring in molecules A and B, respectively. The piperidine ring adopts a chair conformation. This is confirmed by the puckering parameters q_2_ = 0.028 (3) Å, q_3_ = -0.567 (4) Å = Q_T_, δ = 177.2 (4)° for molecule A, and q_2_ = 0.020 (4) Å, q_3_ = -0.569 (4) Å = Q_T_, δ = 178.0 (4)° for molecule B.

The molecular structure is influenced by four intramolecular C—H···O close contacts. The crystal packing is stabilized by weak intermolecular C—H···π interactions (Table 1).

### Experimental

A mixture of *N*-alkenyl-3-methyl indole-2-carbaldehyde (1mmol) and pipecolinic acid (1.3mmol) was heated unde Dean-Stark reflux condition for about 12 hours in toluene (10ml) as solvent. The solvent was removed under vacuo. The crude product was subjected to column chromatography using ethyl acetate and hexane as eleuent in 2:1 ratio. Single crystals were grown by slow evaporation from methanol.

### Refinement

H atoms were placed in idealized positions and allowed to ride on their parent atoms, with C—H distances of 0.93-0.98 Å, and Uiso(H) = 1.5U_eq_(C) for methyl H and Uiso(H) = 1.2U_eq_(C) for all other H atoms.

### Crystal data

**Table:**

C_27_H_30_N_2_O_3_	*F*(000) = 1840
*M**_r_* = 430.53	*D*_x_ = 1.252 Mg m^−^^3^
Monoclinic, *P*2_1_/*n*	Mo *K*α radiation, λ = 0.71073 Å
Hall symbol: -P 2yn	Cell parameters from 22588 reflections
*a* = 22.672 (3) Å	θ = 1.9–27.6°
*b* = 8.8049 (11) Å	µ = 0.08 mm^−^^1^
*c* = 24.431 (3) Å	*T* = 292 K
β = 110.487 (2)°	Block, colourless
*V* = 4568.6 (10) Å^3^	0.22 × 0.20 × 0.18 mm
*Z* = 8	

### Data collection

**Table:**

Bruker SMART APEX CCD area-detector diffractometer	5762 reflections with *I* > 2σ(*I*)
Radiation source: fine-focus sealed tube	*R*_int_ = 0.069
Graphite monochromator	θ_max_ = 25.0°, θ_min_ = 1.1°
ω scans	*h* = −26→26
42435 measured reflections	*k* = −10→10
8045 independent reflections	*l* = −29→29

### Refinement

**Table:**

Refinement on *F*^2^	Primary atom site location: structure-invariant direct methods
Least-squares matrix: full	Secondary atom site location: difference Fourier map
*R*[*F*^2^ > 2σ(*F*^2^)] = 0.092	Hydrogen site location: inferred from neighbouring sites
*wR*(*F*^2^) = 0.182	H-atom parameters constrained
*S* = 1.18	*w* = 1/[σ^2^(*F*_o_^2^) + (0.0508*P*)^2^ + 3.4329*P*] where *P* = (*F*_o_^2^ + 2*F*_c_^2^)/3
8045 reflections	(Δ/σ)_max_ < 0.001
583 parameters	Δρ_max_ = 0.43 e Å^−^^3^
0 restraints	Δρ_min_ = −0.16 e Å^−^^3^

### Special details

**Table:**

Geometry. All esds (except the esd in the dihedral angle between two l.s. planes) are estimated using the full covariance matrix. The cell esds are taken into account individually in the estimation of esds in distances, angles and torsion angles; correlations between esds in cell parameters are only used when they are defined by crystal symmetry. An approximate (isotropic) treatment of cell esds is used for estimating esds involving l.s. planes.
Refinement. Refinement of F^2^ against ALL reflections. The weighted R-factor wR and goodness of fit S are based on F^2^, conventional R-factors R are based on F, with F set to zero for negative F^2^. The threshold expression of F^2^ > 2sigma(F^2^) is used only for calculating R-factors(gt) etc. and is not relevant to the choice of reflections for refinement. R-factors based on F^2^ are statistically about twice as large as those based on F, and R- factors based on ALL data will be even larger.

### Fractional atomic coordinates and isotropic or equivalent isotropic displacement parameters (Å^2^)

**Table:**

	*x*	*y*	*z*	*U*_iso_*/*U*_eq_	
O1A	0.93444 (12)	0.4156 (3)	0.76712 (11)	0.0650 (8)	
O2A	0.97958 (11)	0.2081 (3)	0.74686 (10)	0.0518 (7)	
O3A	0.98712 (14)	0.7518 (3)	0.52518 (13)	0.0684 (8)	
N1A	0.77445 (12)	0.3472 (3)	0.65973 (12)	0.0380 (7)	
N2A	0.85026 (12)	0.0283 (3)	0.63712 (11)	0.0355 (7)	
C1A	0.71633 (15)	0.4049 (4)	0.65603 (14)	0.0395 (8)	
C2A	0.69213 (17)	0.5525 (4)	0.64633 (16)	0.0520 (10)	
H2A	0.7154	0.6324	0.6393	0.062*	
C3A	0.63208 (18)	0.5750 (5)	0.64767 (19)	0.0645 (12)	
H3A	0.6148	0.6720	0.6417	0.077*	
C4A	0.59699 (18)	0.4540 (6)	0.65787 (19)	0.0678 (13)	
H4A	0.5566	0.4724	0.6579	0.081*	
C5A	0.62091 (17)	0.3086 (5)	0.66792 (18)	0.0586 (11)	
H5A	0.5972	0.2301	0.6751	0.070*	
C6A	0.68210 (15)	0.2802 (4)	0.66717 (15)	0.0417 (9)	
C7A	0.72152 (15)	0.1464 (4)	0.67820 (14)	0.0399 (8)	
C8A	0.77736 (15)	0.1932 (4)	0.67319 (13)	0.0345 (8)	
C9A	0.84286 (14)	0.1327 (4)	0.68173 (14)	0.0347 (8)	
H9A	0.8618	0.0883	0.7208	0.042*	
C10A	0.80467 (16)	−0.0964 (4)	0.61825 (16)	0.0445 (9)	
H10A	0.7626	−0.0552	0.5999	0.053*	
H10B	0.8053	−0.1555	0.6519	0.053*	
C11A	0.82090 (17)	−0.1991 (4)	0.57477 (16)	0.0477 (9)	
H11A	0.8605	−0.2507	0.5946	0.057*	
H11B	0.7884	−0.2756	0.5599	0.057*	
C12A	0.82629 (17)	−0.1066 (4)	0.52395 (16)	0.0483 (9)	
H12A	0.7856	−0.0635	0.5016	0.058*	
H12B	0.8391	−0.1724	0.4983	0.058*	
C13A	0.87489 (16)	0.0221 (4)	0.54669 (15)	0.0438 (9)	
H13A	0.9163	−0.0210	0.5662	0.053*	
H13B	0.8764	0.0830	0.5141	0.053*	
C14A	0.85678 (15)	0.1224 (4)	0.58946 (14)	0.0355 (8)	
H14A	0.8170	0.1747	0.5689	0.043*	
C15A	0.90639 (14)	0.2377 (4)	0.62553 (14)	0.0349 (8)	
H15A	0.9454	0.1806	0.6439	0.042*	
C16A	0.88089 (14)	0.2804 (4)	0.67589 (14)	0.0336 (8)	
C17A	0.83276 (14)	0.4144 (4)	0.65852 (15)	0.0383 (8)	
H17A	0.8463	0.4972	0.6863	0.046*	
H17B	0.8276	0.4521	0.6198	0.046*	
C18A	0.70330 (18)	−0.0097 (4)	0.69300 (17)	0.0580 (11)	
H18A	0.6801	−0.0625	0.6576	0.087*	
H18B	0.6776	0.0006	0.7167	0.087*	
H18C	0.7406	−0.0660	0.7140	0.087*	
C19A	0.92336 (14)	0.3740 (4)	0.59520 (14)	0.0347 (8)	
C20A	0.97449 (16)	0.4649 (4)	0.62738 (16)	0.0438 (9)	
H20A	0.9959	0.4422	0.6665	0.053*	
C21A	0.99417 (17)	0.5878 (4)	0.60261 (17)	0.0511 (10)	
H21A	1.0283	0.6458	0.6252	0.061*	
C22A	0.96325 (18)	0.6244 (4)	0.54443 (17)	0.0471 (9)	
C23A	0.91267 (18)	0.5370 (4)	0.51098 (16)	0.0487 (10)	
H23A	0.8918	0.5601	0.4718	0.058*	
C24A	0.89286 (16)	0.4123 (4)	0.53676 (15)	0.0442 (9)	
H24A	0.8587	0.3546	0.5141	0.053*	
C25A	0.9615 (2)	0.7860 (5)	0.46547 (19)	0.0712 (13)	
H25A	0.9657	0.6996	0.4431	0.107*	
H25B	0.9835	0.8709	0.4571	0.107*	
H25C	0.9178	0.8110	0.4553	0.107*	
C26A	0.93398 (15)	0.3123 (4)	0.73493 (15)	0.0369 (8)	
C27A	1.03216 (17)	0.2245 (5)	0.80178 (16)	0.0598 (11)	
H27A	1.0528	0.3199	0.8021	0.090*	
H27B	1.0615	0.1431	0.8057	0.090*	
H27C	1.0168	0.2212	0.8337	0.090*	
O1B	0.92694 (12)	0.3911 (3)	0.24563 (12)	0.0664 (8)	
O2B	0.96821 (11)	0.1798 (3)	0.22344 (10)	0.0506 (7)	
O3B	0.93960 (14)	0.7123 (3)	−0.02072 (13)	0.0714 (8)	
N1B	0.76287 (12)	0.3170 (3)	0.15110 (12)	0.0391 (7)	
N2B	0.83195 (12)	0.0008 (3)	0.11908 (11)	0.0351 (7)	
C1B	0.70797 (15)	0.3754 (4)	0.15541 (16)	0.0437 (9)	
C2B	0.68360 (17)	0.5233 (4)	0.14735 (19)	0.0611 (12)	
H2B	0.7036	0.6020	0.1353	0.073*	
C3B	0.62788 (19)	0.5468 (5)	0.1583 (2)	0.0754 (14)	
H3B	0.6106	0.6439	0.1539	0.090*	
C4B	0.59718 (18)	0.4284 (6)	0.1758 (2)	0.0729 (14)	
H4B	0.5599	0.4483	0.1824	0.087*	
C5B	0.62117 (17)	0.2829 (5)	0.18336 (18)	0.0597 (11)	
H5B	0.6005	0.2053	0.1952	0.072*	
C6B	0.67779 (15)	0.2531 (4)	0.17295 (15)	0.0443 (9)	
C7B	0.71620 (16)	0.1184 (4)	0.17916 (15)	0.0416 (8)	
C8B	0.76781 (15)	0.1646 (4)	0.16583 (14)	0.0357 (8)	
C9B	0.83080 (14)	0.1033 (4)	0.16703 (14)	0.0349 (8)	
H9B	0.8535	0.0569	0.2051	0.042*	
C10B	0.78393 (17)	−0.1183 (4)	0.10156 (16)	0.0476 (9)	
H10C	0.7425	−0.0725	0.0856	0.057*	
H10D	0.7856	−0.1782	0.1354	0.057*	
C11B	0.79522 (19)	−0.2210 (4)	0.05554 (17)	0.0545 (10)	
H11C	0.8341	−0.2770	0.0732	0.065*	
H11D	0.7611	−0.2938	0.0415	0.065*	
C12B	0.79925 (18)	−0.1278 (4)	0.00400 (16)	0.0528 (10)	
H12C	0.8097	−0.1941	−0.0230	0.063*	
H12D	0.7587	−0.0816	−0.0167	0.063*	
C13B	0.84929 (17)	−0.0040 (4)	0.02554 (15)	0.0468 (9)	
H13C	0.8496	0.0579	−0.0072	0.056*	
H13D	0.8905	−0.0503	0.0428	0.056*	
C14B	0.83553 (15)	0.0962 (4)	0.07091 (14)	0.0349 (8)	
H14B	0.7957	0.1503	0.0527	0.042*	
C15B	0.88737 (15)	0.2088 (4)	0.10429 (13)	0.0342 (8)	
H15B	0.9266	0.1507	0.1199	0.041*	
C16B	0.86703 (14)	0.2515 (3)	0.15801 (13)	0.0307 (7)	
C17B	0.81836 (14)	0.3840 (4)	0.14360 (15)	0.0364 (8)	
H17C	0.8341	0.4686	0.1702	0.044*	
H17D	0.8089	0.4194	0.1038	0.044*	
C18B	0.70112 (19)	−0.0372 (5)	0.19660 (18)	0.0625 (11)	
H18D	0.7395	−0.0930	0.2143	0.094*	
H18E	0.6746	−0.0904	0.1626	0.094*	
H18F	0.6797	−0.0271	0.2240	0.094*	
C19B	0.90076 (15)	0.3438 (4)	0.07165 (14)	0.0344 (8)	
C20B	0.95591 (16)	0.4278 (4)	0.09698 (16)	0.0462 (9)	
H20B	0.9834	0.4009	0.1339	0.055*	
C21B	0.97102 (17)	0.5518 (4)	0.06817 (18)	0.0517 (10)	
H21B	1.0079	0.6064	0.0860	0.062*	
C22B	0.93026 (19)	0.5924 (4)	0.01255 (17)	0.0487 (10)	
C24B	0.86112 (16)	0.3885 (4)	0.01548 (15)	0.0430 (9)	
H24B	0.8242	0.3346	−0.0028	0.052*	
C23B	0.87542 (18)	0.5098 (4)	−0.01331 (17)	0.0498 (10)	
H23B	0.8481	0.5365	−0.0503	0.060*	
C25B	0.9953 (2)	0.7948 (5)	0.0030 (2)	0.0790 (14)	
H25D	0.9966	0.8406	0.0391	0.119*	
H25E	0.9969	0.8726	−0.0240	0.119*	
H25F	1.0306	0.7280	0.0102	0.119*	
C26B	0.92342 (15)	0.2853 (4)	0.21397 (14)	0.0352 (8)	
C27B	1.02450 (16)	0.1965 (5)	0.27538 (16)	0.0559 (11)	
H27D	1.0443	0.2920	0.2738	0.084*	
H27E	1.0532	0.1153	0.2769	0.084*	
H27F	1.0130	0.1934	0.3096	0.084*	

### Atomic displacement parameters (Å^2^)

**Table:**

	*U*^11^	*U*^22^	*U*^33^	*U*^12^	*U*^13^	*U*^23^
O1A	0.0552 (17)	0.070 (2)	0.0578 (17)	0.0105 (14)	0.0049 (14)	−0.0295 (16)
O2A	0.0458 (15)	0.0482 (15)	0.0457 (15)	0.0134 (12)	−0.0039 (12)	−0.0069 (12)
O3A	0.081 (2)	0.0602 (19)	0.070 (2)	−0.0084 (16)	0.0342 (17)	0.0040 (16)
N1A	0.0334 (15)	0.0378 (17)	0.0443 (17)	0.0014 (13)	0.0156 (13)	0.0012 (13)
N2A	0.0345 (15)	0.0307 (15)	0.0398 (16)	0.0016 (12)	0.0110 (13)	0.0006 (13)
C1A	0.0300 (18)	0.049 (2)	0.039 (2)	0.0025 (17)	0.0108 (15)	−0.0070 (17)
C2A	0.041 (2)	0.049 (2)	0.063 (3)	0.0056 (18)	0.0156 (19)	−0.005 (2)
C3A	0.047 (2)	0.062 (3)	0.082 (3)	0.018 (2)	0.020 (2)	−0.012 (2)
C4A	0.034 (2)	0.087 (3)	0.082 (3)	0.007 (2)	0.020 (2)	−0.020 (3)
C5A	0.042 (2)	0.071 (3)	0.067 (3)	−0.008 (2)	0.025 (2)	−0.017 (2)
C6A	0.0327 (19)	0.053 (2)	0.039 (2)	−0.0047 (17)	0.0128 (16)	−0.0109 (17)
C7A	0.0371 (19)	0.046 (2)	0.0371 (19)	−0.0065 (17)	0.0141 (16)	−0.0050 (16)
C8A	0.0361 (19)	0.0356 (19)	0.0307 (18)	0.0001 (15)	0.0104 (15)	0.0004 (15)
C9A	0.0341 (18)	0.0356 (19)	0.0303 (18)	−0.0006 (15)	0.0060 (14)	0.0031 (15)
C10A	0.041 (2)	0.034 (2)	0.056 (2)	−0.0012 (16)	0.0137 (17)	0.0034 (17)
C11A	0.048 (2)	0.032 (2)	0.062 (2)	0.0013 (17)	0.0182 (19)	−0.0066 (18)
C12A	0.052 (2)	0.041 (2)	0.052 (2)	0.0029 (18)	0.0190 (19)	−0.0090 (18)
C13A	0.046 (2)	0.040 (2)	0.048 (2)	0.0000 (17)	0.0200 (18)	−0.0058 (17)
C14A	0.0315 (17)	0.0296 (18)	0.043 (2)	0.0067 (14)	0.0103 (15)	0.0036 (15)
C15A	0.0276 (17)	0.0362 (19)	0.0375 (19)	0.0048 (14)	0.0072 (15)	−0.0016 (15)
C16A	0.0299 (17)	0.0343 (19)	0.0355 (19)	0.0032 (14)	0.0103 (15)	0.0007 (15)
C17A	0.0371 (19)	0.0348 (19)	0.044 (2)	0.0015 (16)	0.0158 (16)	0.0035 (16)
C18A	0.061 (3)	0.063 (3)	0.059 (3)	−0.013 (2)	0.032 (2)	0.002 (2)
C19A	0.0334 (18)	0.0349 (19)	0.040 (2)	0.0057 (15)	0.0181 (16)	−0.0003 (15)
C20A	0.039 (2)	0.048 (2)	0.047 (2)	−0.0026 (17)	0.0172 (17)	−0.0028 (18)
C21A	0.045 (2)	0.055 (2)	0.054 (2)	−0.0137 (19)	0.0173 (19)	−0.006 (2)
C22A	0.054 (2)	0.035 (2)	0.065 (3)	−0.0057 (18)	0.036 (2)	−0.0042 (19)
C23A	0.059 (2)	0.051 (2)	0.039 (2)	0.011 (2)	0.0213 (19)	0.0052 (18)
C24A	0.044 (2)	0.042 (2)	0.048 (2)	−0.0015 (17)	0.0167 (18)	−0.0022 (18)
C25A	0.082 (3)	0.073 (3)	0.068 (3)	0.012 (3)	0.038 (3)	0.006 (2)
C26A	0.0383 (19)	0.0356 (19)	0.038 (2)	−0.0034 (16)	0.0155 (16)	−0.0038 (17)
C27A	0.046 (2)	0.065 (3)	0.051 (2)	0.005 (2)	−0.0055 (19)	−0.002 (2)
O1B	0.0539 (17)	0.0695 (19)	0.0602 (18)	0.0126 (14)	0.0005 (14)	−0.0304 (16)
O2B	0.0404 (14)	0.0463 (15)	0.0492 (15)	0.0112 (12)	−0.0044 (12)	−0.0073 (12)
O3B	0.081 (2)	0.0570 (18)	0.085 (2)	−0.0100 (16)	0.0414 (18)	0.0069 (16)
N1B	0.0349 (16)	0.0312 (15)	0.0547 (18)	−0.0003 (13)	0.0200 (14)	−0.0019 (14)
N2B	0.0373 (15)	0.0289 (15)	0.0383 (16)	−0.0018 (12)	0.0123 (13)	−0.0001 (12)
C1B	0.0329 (19)	0.046 (2)	0.052 (2)	−0.0016 (17)	0.0152 (17)	−0.0119 (18)
C2B	0.042 (2)	0.046 (2)	0.094 (3)	0.0064 (19)	0.023 (2)	−0.006 (2)
C3B	0.049 (3)	0.056 (3)	0.122 (4)	0.011 (2)	0.032 (3)	−0.019 (3)
C4B	0.037 (2)	0.087 (4)	0.102 (4)	−0.003 (2)	0.033 (2)	−0.035 (3)
C5B	0.040 (2)	0.075 (3)	0.072 (3)	−0.015 (2)	0.029 (2)	−0.023 (2)
C6B	0.0335 (19)	0.054 (2)	0.046 (2)	−0.0097 (17)	0.0136 (17)	−0.0160 (18)
C7B	0.040 (2)	0.045 (2)	0.042 (2)	−0.0105 (17)	0.0160 (16)	−0.0053 (17)
C8B	0.0401 (19)	0.0333 (19)	0.0322 (18)	−0.0020 (15)	0.0108 (15)	−0.0017 (15)
C9B	0.0349 (18)	0.0367 (19)	0.0307 (18)	0.0014 (15)	0.0084 (14)	0.0035 (15)
C10B	0.055 (2)	0.037 (2)	0.051 (2)	−0.0055 (18)	0.0186 (19)	0.0014 (18)
C11B	0.064 (3)	0.035 (2)	0.060 (3)	−0.0080 (19)	0.017 (2)	−0.0101 (19)
C12B	0.061 (2)	0.044 (2)	0.049 (2)	−0.0004 (19)	0.0144 (19)	−0.0126 (19)
C13B	0.051 (2)	0.046 (2)	0.044 (2)	0.0018 (18)	0.0187 (18)	−0.0018 (18)
C14B	0.0333 (18)	0.0342 (19)	0.0361 (19)	0.0044 (15)	0.0107 (15)	0.0039 (15)
C15B	0.0299 (17)	0.0361 (19)	0.0353 (19)	0.0037 (15)	0.0099 (15)	−0.0002 (15)
C16B	0.0296 (17)	0.0290 (17)	0.0333 (18)	0.0003 (14)	0.0107 (14)	0.0014 (14)
C17B	0.0356 (18)	0.0336 (19)	0.041 (2)	0.0009 (15)	0.0147 (16)	0.0010 (15)
C18B	0.064 (3)	0.064 (3)	0.069 (3)	−0.015 (2)	0.034 (2)	0.002 (2)
C19B	0.0336 (18)	0.0346 (19)	0.0402 (19)	0.0016 (15)	0.0194 (16)	−0.0021 (15)
C20B	0.042 (2)	0.052 (2)	0.049 (2)	−0.0036 (18)	0.0223 (18)	−0.0043 (19)
C21B	0.044 (2)	0.048 (2)	0.073 (3)	−0.0146 (18)	0.033 (2)	−0.014 (2)
C22B	0.067 (3)	0.036 (2)	0.058 (3)	0.0089 (19)	0.041 (2)	0.0094 (19)
C24B	0.043 (2)	0.045 (2)	0.043 (2)	−0.0002 (17)	0.0173 (17)	0.0028 (17)
C23B	0.055 (2)	0.047 (2)	0.050 (2)	0.003 (2)	0.021 (2)	0.0067 (19)
C25B	0.085 (3)	0.058 (3)	0.110 (4)	−0.012 (3)	0.054 (3)	−0.013 (3)
C26B	0.0339 (19)	0.036 (2)	0.038 (2)	−0.0015 (16)	0.0150 (16)	−0.0026 (16)
C27B	0.039 (2)	0.060 (3)	0.053 (2)	0.0072 (19)	−0.0042 (18)	−0.001 (2)

### Geometric parameters (Å, º)

**Table:**

O1A—C26A	1.200 (4)	O1B—C26B	1.195 (4)
O2A—C26A	1.336 (4)	O2B—C26B	1.336 (4)
O2A—C27A	1.457 (4)	O2B—C27B	1.459 (4)
O3A—C22A	1.396 (4)	O3B—C22B	1.393 (4)
O3A—C25A	1.401 (5)	O3B—C25B	1.395 (5)
N1A—C1A	1.385 (4)	N1B—C8B	1.384 (4)
N1A—C8A	1.391 (4)	N1B—C1B	1.384 (4)
N1A—C17A	1.458 (4)	N1B—C17B	1.458 (4)
N2A—C10A	1.467 (4)	N2B—C10B	1.463 (4)
N2A—C14A	1.479 (4)	N2B—C14B	1.471 (4)
N2A—C9A	1.479 (4)	N2B—C9B	1.486 (4)
C1A—C2A	1.399 (5)	C1B—C2B	1.402 (5)
C1A—C6A	1.424 (5)	C1B—C6B	1.420 (5)
C2A—C3A	1.387 (5)	C2B—C3B	1.395 (5)
C2A—H2A	0.9300	C2B—H2B	0.9300
C3A—C4A	1.403 (6)	C3B—C4B	1.400 (6)
C3A—H3A	0.9300	C3B—H3B	0.9300
C4A—C5A	1.379 (6)	C4B—C5B	1.379 (6)
C4A—H4A	0.9300	C4B—H4B	0.9300
C5A—C6A	1.416 (5)	C5B—C6B	1.418 (5)
C5A—H5A	0.9300	C5B—H5B	0.9300
C6A—C7A	1.446 (5)	C6B—C7B	1.447 (5)
C7A—C8A	1.377 (4)	C7B—C8B	1.381 (4)
C7A—C18A	1.515 (5)	C7B—C18B	1.509 (5)
C8A—C9A	1.522 (4)	C8B—C9B	1.518 (4)
C9A—C16A	1.594 (4)	C9B—C16B	1.598 (4)
C9A—H9A	0.9800	C9B—H9B	0.9800
C10A—C11A	1.535 (5)	C10B—C11B	1.532 (5)
C10A—H10A	0.9700	C10B—H10C	0.9700
C10A—H10B	0.9700	C10B—H10D	0.9700
C11A—C12A	1.525 (5)	C11B—C12B	1.532 (5)
C11A—H11A	0.9700	C11B—H11C	0.9700
C11A—H11B	0.9700	C11B—H11D	0.9700
C12A—C13A	1.542 (5)	C12B—C13B	1.528 (5)
C12A—H12A	0.9700	C12B—H12C	0.9700
C12A—H12B	0.9700	C12B—H12D	0.9700
C13A—C14A	1.530 (4)	C13B—C14B	1.532 (4)
C13A—H13A	0.9700	C13B—H13C	0.9700
C13A—H13B	0.9700	C13B—H13D	0.9700
C14A—C15A	1.542 (4)	C14B—C15B	1.536 (4)
C14A—H14A	0.9800	C14B—H14B	0.9800
C15A—C19A	1.528 (4)	C15B—C19B	1.520 (4)
C15A—C16A	1.577 (4)	C15B—C16B	1.581 (4)
C15A—H15A	0.9800	C15B—H15B	0.9800
C16A—C26A	1.547 (4)	C16B—C26B	1.539 (4)
C16A—C17A	1.562 (4)	C16B—C17B	1.559 (4)
C17A—H17A	0.9700	C17B—H17C	0.9700
C17A—H17B	0.9700	C17B—H17D	0.9700
C18A—H18A	0.9600	C18B—H18D	0.9600
C18A—H18B	0.9600	C18B—H18E	0.9600
C18A—H18C	0.9600	C18B—H18F	0.9600
C19A—C24A	1.393 (5)	C19B—C20B	1.397 (4)
C19A—C20A	1.402 (5)	C19B—C24B	1.408 (5)
C20A—C21A	1.387 (5)	C20B—C21B	1.405 (5)
C20A—H20A	0.9300	C20B—H20B	0.9300
C21A—C22A	1.385 (5)	C21B—C22B	1.396 (5)
C21A—H21A	0.9300	C21B—H21B	0.9300
C22A—C23A	1.385 (5)	C22B—C23B	1.387 (5)
C23A—C24A	1.415 (5)	C24B—C23B	1.378 (5)
C23A—H23A	0.9300	C24B—H24B	0.9300
C24A—H24A	0.9300	C23B—H23B	0.9300
C25A—H25A	0.9600	C25B—H25D	0.9600
C25A—H25B	0.9600	C25B—H25E	0.9600
C25A—H25C	0.9600	C25B—H25F	0.9600
C27A—H27A	0.9600	C27B—H27D	0.9600
C27A—H27B	0.9600	C27B—H27E	0.9600
C27A—H27C	0.9600	C27B—H27F	0.9600
			
C26A—O2A—C27A	117.0 (3)	C26B—O2B—C27B	117.4 (3)
C22A—O3A—C25A	117.1 (3)	C22B—O3B—C25B	116.9 (4)
C1A—N1A—C8A	109.7 (3)	C8B—N1B—C1B	109.5 (3)
C1A—N1A—C17A	134.3 (3)	C8B—N1B—C17B	115.5 (3)
C8A—N1A—C17A	115.6 (3)	C1B—N1B—C17B	134.2 (3)
C10A—N2A—C14A	115.2 (3)	C10B—N2B—C14B	114.8 (3)
C10A—N2A—C9A	116.6 (3)	C10B—N2B—C9B	116.7 (3)
C14A—N2A—C9A	107.5 (2)	C14B—N2B—C9B	107.7 (2)
N1A—C1A—C2A	131.3 (3)	N1B—C1B—C2B	130.9 (3)
N1A—C1A—C6A	106.0 (3)	N1B—C1B—C6B	106.4 (3)
C2A—C1A—C6A	122.7 (3)	C2B—C1B—C6B	122.6 (3)
C3A—C2A—C1A	117.3 (4)	C3B—C2B—C1B	116.7 (4)
C3A—C2A—H2A	121.4	C3B—C2B—H2B	121.6
C1A—C2A—H2A	121.4	C1B—C2B—H2B	121.6
C2A—C3A—C4A	121.2 (4)	C2B—C3B—C4B	121.9 (4)
C2A—C3A—H3A	119.4	C2B—C3B—H3B	119.1
C4A—C3A—H3A	119.4	C4B—C3B—H3B	119.1
C5A—C4A—C3A	121.6 (4)	C5B—C4B—C3B	121.2 (4)
C5A—C4A—H4A	119.2	C5B—C4B—H4B	119.4
C3A—C4A—H4A	119.2	C3B—C4B—H4B	119.4
C4A—C5A—C6A	119.2 (4)	C4B—C5B—C6B	119.2 (4)
C4A—C5A—H5A	120.4	C4B—C5B—H5B	120.4
C6A—C5A—H5A	120.4	C6B—C5B—H5B	120.4
C5A—C6A—C1A	117.9 (3)	C5B—C6B—C1B	118.4 (3)
C5A—C6A—C7A	133.3 (3)	C5B—C6B—C7B	133.0 (4)
C1A—C6A—C7A	108.7 (3)	C1B—C6B—C7B	108.5 (3)
C8A—C7A—C6A	105.5 (3)	C8B—C7B—C6B	105.2 (3)
C8A—C7A—C18A	129.0 (3)	C8B—C7B—C18B	128.9 (3)
C6A—C7A—C18A	125.5 (3)	C6B—C7B—C18B	125.9 (3)
C7A—C8A—N1A	110.1 (3)	C7B—C8B—N1B	110.4 (3)
C7A—C8A—C9A	140.0 (3)	C7B—C8B—C9B	139.5 (3)
N1A—C8A—C9A	109.7 (3)	N1B—C8B—C9B	109.9 (3)
N2A—C9A—C8A	118.3 (3)	N2B—C9B—C8B	118.5 (3)
N2A—C9A—C16A	103.7 (2)	N2B—C9B—C16B	103.6 (2)
C8A—C9A—C16A	103.5 (2)	C8B—C9B—C16B	103.4 (2)
N2A—C9A—H9A	110.3	N2B—C9B—H9B	110.2
C8A—C9A—H9A	110.3	C8B—C9B—H9B	110.2
C16A—C9A—H9A	110.3	C16B—C9B—H9B	110.2
N2A—C10A—C11A	110.2 (3)	N2B—C10B—C11B	109.8 (3)
N2A—C10A—H10A	109.6	N2B—C10B—H10C	109.7
C11A—C10A—H10A	109.6	C11B—C10B—H10C	109.7
N2A—C10A—H10B	109.6	N2B—C10B—H10D	109.7
C11A—C10A—H10B	109.6	C11B—C10B—H10D	109.7
H10A—C10A—H10B	108.1	H10C—C10B—H10D	108.2
C12A—C11A—C10A	110.8 (3)	C12B—C11B—C10B	111.1 (3)
C12A—C11A—H11A	109.5	C12B—C11B—H11C	109.4
C10A—C11A—H11A	109.5	C10B—C11B—H11C	109.4
C12A—C11A—H11B	109.5	C12B—C11B—H11D	109.4
C10A—C11A—H11B	109.5	C10B—C11B—H11D	109.4
H11A—C11A—H11B	108.1	H11C—C11B—H11D	108.0
C11A—C12A—C13A	110.5 (3)	C13B—C12B—C11B	110.5 (3)
C11A—C12A—H12A	109.6	C13B—C12B—H12C	109.6
C13A—C12A—H12A	109.6	C11B—C12B—H12C	109.6
C11A—C12A—H12B	109.6	C13B—C12B—H12D	109.6
C13A—C12A—H12B	109.6	C11B—C12B—H12D	109.6
H12A—C12A—H12B	108.1	H12C—C12B—H12D	108.1
C14A—C13A—C12A	110.0 (3)	C12B—C13B—C14B	110.2 (3)
C14A—C13A—H13A	109.7	C12B—C13B—H13C	109.6
C12A—C13A—H13A	109.7	C14B—C13B—H13C	109.6
C14A—C13A—H13B	109.7	C12B—C13B—H13D	109.6
C12A—C13A—H13B	109.7	C14B—C13B—H13D	109.6
H13A—C13A—H13B	108.2	H13C—C13B—H13D	108.1
N2A—C14A—C13A	109.8 (3)	N2B—C14B—C13B	109.4 (3)
N2A—C14A—C15A	100.0 (2)	N2B—C14B—C15B	101.0 (2)
C13A—C14A—C15A	117.0 (3)	C13B—C14B—C15B	116.4 (3)
N2A—C14A—H14A	109.9	N2B—C14B—H14B	109.9
C13A—C14A—H14A	109.9	C13B—C14B—H14B	109.9
C15A—C14A—H14A	109.9	C15B—C14B—H14B	109.9
C19A—C15A—C14A	119.8 (3)	C19B—C15B—C14B	118.8 (3)
C19A—C15A—C16A	114.5 (3)	C19B—C15B—C16B	114.8 (3)
C14A—C15A—C16A	102.1 (2)	C14B—C15B—C16B	101.7 (2)
C19A—C15A—H15A	106.5	C19B—C15B—H15B	106.9
C14A—C15A—H15A	106.5	C14B—C15B—H15B	106.9
C16A—C15A—H15A	106.5	C16B—C15B—H15B	106.9
C26A—C16A—C17A	111.0 (3)	C26B—C16B—C17B	111.0 (3)
C26A—C16A—C15A	113.1 (2)	C26B—C16B—C15B	113.0 (2)
C17A—C16A—C15A	112.3 (3)	C17B—C16B—C15B	112.3 (3)
C26A—C16A—C9A	109.5 (2)	C26B—C16B—C9B	110.0 (2)
C17A—C16A—C9A	107.0 (2)	C17B—C16B—C9B	106.6 (2)
C15A—C16A—C9A	103.5 (2)	C15B—C16B—C9B	103.5 (2)
N1A—C17A—C16A	103.9 (3)	N1B—C17B—C16B	104.0 (2)
N1A—C17A—H17A	111.0	N1B—C17B—H17C	111.0
C16A—C17A—H17A	111.0	C16B—C17B—H17C	111.0
N1A—C17A—H17B	111.0	N1B—C17B—H17D	111.0
C16A—C17A—H17B	111.0	C16B—C17B—H17D	111.0
H17A—C17A—H17B	109.0	H17C—C17B—H17D	109.0
C7A—C18A—H18A	109.5	C7B—C18B—H18D	109.5
C7A—C18A—H18B	109.5	C7B—C18B—H18E	109.5
H18A—C18A—H18B	109.5	H18D—C18B—H18E	109.5
C7A—C18A—H18C	109.5	C7B—C18B—H18F	109.5
H18A—C18A—H18C	109.5	H18D—C18B—H18F	109.5
H18B—C18A—H18C	109.5	H18E—C18B—H18F	109.5
C24A—C19A—C20A	116.9 (3)	C20B—C19B—C24B	116.8 (3)
C24A—C19A—C15A	124.6 (3)	C20B—C19B—C15B	119.6 (3)
C20A—C19A—C15A	118.4 (3)	C24B—C19B—C15B	123.6 (3)
C21A—C20A—C19A	122.0 (3)	C19B—C20B—C21B	121.8 (4)
C21A—C20A—H20A	119.0	C19B—C20B—H20B	119.1
C19A—C20A—H20A	119.0	C21B—C20B—H20B	119.1
C22A—C21A—C20A	120.3 (3)	C22B—C21B—C20B	119.5 (3)
C22A—C21A—H21A	119.8	C22B—C21B—H21B	120.2
C20A—C21A—H21A	119.8	C20B—C21B—H21B	120.2
C21A—C22A—C23A	119.5 (3)	C23B—C22B—O3B	115.2 (4)
C21A—C22A—O3A	114.6 (3)	C23B—C22B—C21B	119.4 (3)
C23A—C22A—O3A	125.9 (4)	O3B—C22B—C21B	125.3 (4)
C22A—C23A—C24A	119.6 (3)	C23B—C24B—C19B	122.0 (3)
C22A—C23A—H23A	120.2	C23B—C24B—H24B	119.0
C24A—C23A—H23A	120.2	C19B—C24B—H24B	119.0
C19A—C24A—C23A	121.6 (3)	C24B—C23B—C22B	120.5 (4)
C19A—C24A—H24A	119.2	C24B—C23B—H23B	119.8
C23A—C24A—H24A	119.2	C22B—C23B—H23B	119.8
O3A—C25A—H25A	109.5	O3B—C25B—H25D	109.5
O3A—C25A—H25B	109.5	O3B—C25B—H25E	109.5
H25A—C25A—H25B	109.5	H25D—C25B—H25E	109.5
O3A—C25A—H25C	109.5	O3B—C25B—H25F	109.5
H25A—C25A—H25C	109.5	H25D—C25B—H25F	109.5
H25B—C25A—H25C	109.5	H25E—C25B—H25F	109.5
O1A—C26A—O2A	123.3 (3)	O1B—C26B—O2B	123.7 (3)
O1A—C26A—C16A	125.0 (3)	O1B—C26B—C16B	124.9 (3)
O2A—C26A—C16A	111.6 (3)	O2B—C26B—C16B	111.3 (3)
O2A—C27A—H27A	109.5	O2B—C27B—H27D	109.5
O2A—C27A—H27B	109.5	O2B—C27B—H27E	109.5
H27A—C27A—H27B	109.5	H27D—C27B—H27E	109.5
O2A—C27A—H27C	109.5	O2B—C27B—H27F	109.5
H27A—C27A—H27C	109.5	H27D—C27B—H27F	109.5
H27B—C27A—H27C	109.5	H27E—C27B—H27F	109.5
			
C8A—N1A—C1A—C2A	176.7 (4)	C8B—N1B—C1B—C2B	177.2 (4)
C17A—N1A—C1A—C2A	4.1 (6)	C17B—N1B—C1B—C2B	8.2 (7)
C8A—N1A—C1A—C6A	−0.6 (4)	C8B—N1B—C1B—C6B	−0.6 (4)
C17A—N1A—C1A—C6A	−173.2 (3)	C17B—N1B—C1B—C6B	−169.6 (3)
N1A—C1A—C2A—C3A	−177.2 (4)	N1B—C1B—C2B—C3B	−176.5 (4)
C6A—C1A—C2A—C3A	−0.3 (5)	C6B—C1B—C2B—C3B	1.0 (6)
C1A—C2A—C3A—C4A	−0.5 (6)	C1B—C2B—C3B—C4B	−0.8 (7)
C2A—C3A—C4A—C5A	1.1 (7)	C2B—C3B—C4B—C5B	0.5 (7)
C3A—C4A—C5A—C6A	−0.8 (6)	C3B—C4B—C5B—C6B	−0.3 (7)
C4A—C5A—C6A—C1A	0.1 (5)	C4B—C5B—C6B—C1B	0.5 (6)
C4A—C5A—C6A—C7A	176.9 (4)	C4B—C5B—C6B—C7B	176.8 (4)
N1A—C1A—C6A—C5A	178.0 (3)	N1B—C1B—C6B—C5B	177.2 (3)
C2A—C1A—C6A—C5A	0.5 (5)	C2B—C1B—C6B—C5B	−0.8 (6)
N1A—C1A—C6A—C7A	0.5 (4)	N1B—C1B—C6B—C7B	0.0 (4)
C2A—C1A—C6A—C7A	−177.1 (3)	C2B—C1B—C6B—C7B	−178.0 (3)
C5A—C6A—C7A—C8A	−177.3 (4)	C5B—C6B—C7B—C8B	−176.0 (4)
C1A—C6A—C7A—C8A	−0.3 (4)	C1B—C6B—C7B—C8B	0.6 (4)
C5A—C6A—C7A—C18A	1.8 (6)	C5B—C6B—C7B—C18B	4.0 (6)
C1A—C6A—C7A—C18A	178.8 (3)	C1B—C6B—C7B—C18B	−179.4 (3)
C6A—C7A—C8A—N1A	−0.1 (4)	C6B—C7B—C8B—N1B	−0.9 (4)
C18A—C7A—C8A—N1A	−179.1 (3)	C18B—C7B—C8B—N1B	179.0 (3)
C6A—C7A—C8A—C9A	174.3 (4)	C6B—C7B—C8B—C9B	172.5 (4)
C18A—C7A—C8A—C9A	−4.7 (7)	C18B—C7B—C8B—C9B	−7.5 (7)
C1A—N1A—C8A—C7A	0.4 (4)	C1B—N1B—C8B—C7B	1.0 (4)
C17A—N1A—C8A—C7A	174.5 (3)	C17B—N1B—C8B—C7B	172.2 (3)
C1A—N1A—C8A—C9A	−175.8 (3)	C1B—N1B—C8B—C9B	−174.5 (3)
C17A—N1A—C8A—C9A	−1.6 (4)	C17B—N1B—C8B—C9B	−3.2 (4)
C10A—N2A—C9A—C8A	−46.8 (4)	C10B—N2B—C9B—C8B	−44.1 (4)
C14A—N2A—C9A—C8A	84.2 (3)	C14B—N2B—C9B—C8B	86.6 (3)
C10A—N2A—C9A—C16A	−160.7 (3)	C10B—N2B—C9B—C16B	−157.9 (3)
C14A—N2A—C9A—C16A	−29.7 (3)	C14B—N2B—C9B—C16B	−27.1 (3)
C7A—C8A—C9A—N2A	75.8 (5)	C7B—C8B—C9B—N2B	79.4 (5)
N1A—C8A—C9A—N2A	−109.7 (3)	N1B—C8B—C9B—N2B	−107.2 (3)
C7A—C8A—C9A—C16A	−170.2 (4)	C7B—C8B—C9B—C16B	−166.7 (4)
N1A—C8A—C9A—C16A	4.2 (3)	N1B—C8B—C9B—C16B	6.7 (3)
C14A—N2A—C10A—C11A	55.9 (4)	C14B—N2B—C10B—C11B	57.3 (4)
C9A—N2A—C10A—C11A	−176.8 (3)	C9B—N2B—C10B—C11B	−175.3 (3)
N2A—C10A—C11A—C12A	−54.1 (4)	N2B—C10B—C11B—C12B	−53.9 (4)
C10A—C11A—C12A—C13A	55.8 (4)	C10B—C11B—C12B—C13B	54.7 (4)
C11A—C12A—C13A—C14A	−56.6 (4)	C11B—C12B—C13B—C14B	−55.9 (4)
C10A—N2A—C14A—C13A	−57.1 (3)	C10B—N2B—C14B—C13B	−58.8 (3)
C9A—N2A—C14A—C13A	171.2 (2)	C9B—N2B—C14B—C13B	169.4 (2)
C10A—N2A—C14A—C15A	179.4 (2)	C10B—N2B—C14B—C15B	178.0 (3)
C9A—N2A—C14A—C15A	47.6 (3)	C9B—N2B—C14B—C15B	46.2 (3)
C12A—C13A—C14A—N2A	55.6 (4)	C12B—C13B—C14B—N2B	56.5 (4)
C12A—C13A—C14A—C15A	168.5 (3)	C12B—C13B—C14B—C15B	170.0 (3)
N2A—C14A—C15A—C19A	−172.6 (3)	N2B—C14B—C15B—C19B	−172.0 (3)
C13A—C14A—C15A—C19A	69.0 (4)	C13B—C14B—C15B—C19B	69.7 (4)
N2A—C14A—C15A—C16A	−44.9 (3)	N2B—C14B—C15B—C16B	−44.9 (3)
C13A—C14A—C15A—C16A	−163.3 (3)	C13B—C14B—C15B—C16B	−163.2 (3)
C19A—C15A—C16A—C26A	−83.2 (3)	C19B—C15B—C16B—C26B	−82.8 (3)
C14A—C15A—C16A—C26A	145.8 (3)	C14B—C15B—C16B—C26B	147.6 (3)
C19A—C15A—C16A—C17A	43.4 (4)	C19B—C15B—C16B—C17B	43.7 (3)
C14A—C15A—C16A—C17A	−87.6 (3)	C14B—C15B—C16B—C17B	−86.0 (3)
C19A—C15A—C16A—C9A	158.4 (2)	C19B—C15B—C16B—C9B	158.3 (3)
C14A—C15A—C16A—C9A	27.5 (3)	C14B—C15B—C16B—C9B	28.6 (3)
N2A—C9A—C16A—C26A	−120.8 (3)	N2B—C9B—C16B—C26B	−123.1 (3)
C8A—C9A—C16A—C26A	115.1 (3)	C8B—C9B—C16B—C26B	112.7 (3)
N2A—C9A—C16A—C17A	118.9 (3)	N2B—C9B—C16B—C17B	116.5 (3)
C8A—C9A—C16A—C17A	−5.2 (3)	C8B—C9B—C16B—C17B	−7.7 (3)
N2A—C9A—C16A—C15A	0.1 (3)	N2B—C9B—C16B—C15B	−2.1 (3)
C8A—C9A—C16A—C15A	−124.0 (2)	C8B—C9B—C16B—C15B	−126.3 (2)
C1A—N1A—C17A—C16A	170.5 (3)	C8B—N1B—C17B—C16B	−2.0 (4)
C8A—N1A—C17A—C16A	−1.8 (4)	C1B—N1B—C17B—C16B	166.4 (3)
C26A—C16A—C17A—N1A	−115.1 (3)	C26B—C16B—C17B—N1B	−113.7 (3)
C15A—C16A—C17A—N1A	117.2 (3)	C15B—C16B—C17B—N1B	118.7 (3)
C9A—C16A—C17A—N1A	4.3 (3)	C9B—C16B—C17B—N1B	6.1 (3)
C14A—C15A—C19A—C24A	6.4 (5)	C14B—C15B—C19B—C20B	−165.9 (3)
C16A—C15A—C19A—C24A	−115.3 (3)	C16B—C15B—C19B—C20B	73.5 (4)
C14A—C15A—C19A—C20A	−171.4 (3)	C14B—C15B—C19B—C24B	12.3 (5)
C16A—C15A—C19A—C20A	66.9 (4)	C16B—C15B—C19B—C24B	−108.4 (3)
C24A—C19A—C20A—C21A	0.0 (5)	C24B—C19B—C20B—C21B	0.5 (5)
C15A—C19A—C20A—C21A	178.0 (3)	C15B—C19B—C20B—C21B	178.8 (3)
C19A—C20A—C21A—C22A	0.0 (5)	C19B—C20B—C21B—C22B	−0.4 (5)
C20A—C21A—C22A—C23A	−0.4 (5)	C25B—O3B—C22B—C23B	−177.9 (3)
C20A—C21A—C22A—O3A	179.1 (3)	C25B—O3B—C22B—C21B	2.2 (5)
C25A—O3A—C22A—C21A	174.3 (3)	C20B—C21B—C22B—C23B	0.1 (5)
C25A—O3A—C22A—C23A	−6.3 (5)	C20B—C21B—C22B—O3B	−179.9 (3)
C21A—C22A—C23A—C24A	0.6 (5)	C20B—C19B—C24B—C23B	−0.4 (5)
O3A—C22A—C23A—C24A	−178.8 (3)	C15B—C19B—C24B—C23B	−178.7 (3)
C20A—C19A—C24A—C23A	0.2 (5)	C19B—C24B—C23B—C22B	0.2 (5)
C15A—C19A—C24A—C23A	−177.6 (3)	O3B—C22B—C23B—C24B	180.0 (3)
C22A—C23A—C24A—C19A	−0.5 (5)	C21B—C22B—C23B—C24B	0.0 (5)
C27A—O2A—C26A—O1A	−0.6 (5)	C27B—O2B—C26B—O1B	−0.4 (5)
C27A—O2A—C26A—C16A	−179.3 (3)	C27B—O2B—C26B—C16B	−179.6 (3)
C17A—C16A—C26A—O1A	9.1 (5)	C17B—C16B—C26B—O1B	7.0 (5)
C15A—C16A—C26A—O1A	136.4 (4)	C15B—C16B—C26B—O1B	134.2 (4)
C9A—C16A—C26A—O1A	−108.8 (4)	C9B—C16B—C26B—O1B	−110.7 (4)
C17A—C16A—C26A—O2A	−172.2 (3)	C17B—C16B—C26B—O2B	−173.8 (3)
C15A—C16A—C26A—O2A	−44.9 (4)	C15B—C16B—C26B—O2B	−46.6 (4)
C9A—C16A—C26A—O2A	69.9 (3)	C9B—C16B—C26B—O2B	68.5 (3)

### Hydrogen-bond geometry (Å, º)

Cg1 and Cg2 are the centroids of the C19A–C24A and C19B–C24B rings, respectively.

**Table:**

*D*—H···*A*	*D*—H	H···*A*	*D*···*A*	*D*—H···*A*
C17*B*—H17*C*···O1*B*	0.97	2.36	2.825 (4)	109
C17*A*—H17*A*···O1*A*	0.97	2.38	2.841 (4)	109
C15*B*—H15*B*···O2*B*	0.98	2.39	2.857 (4)	109
C15*A*—H15*A*···O2*A*	0.98	2.37	2.858 (4)	110
C25*A*—H25*A*···*Cg*1^i^	0.96	2.81	3.606 (5)	141
C25*B*—H25*F*···*Cg*2^ii^	0.96	2.68	3.495 (5)	143

Symmetry codes: (i) −*x*+2, −*y*+1, −*z*+1; (ii) −*x*+2, −*y*+1, −*z*.
